# Ceramic art design generation and aesthetic quality evaluation based on multimodal large language models and diffusion probabilistic framework

**DOI:** 10.1038/s41598-026-55502-z

**Published:** 2026-06-11

**Authors:** Wenda Zhao

**Affiliations:** College of ART, Hanjiang Normal University, Shiyan, 442000 Hubei China

**Keywords:** Ceramic art design, Multimodal large language models, Diffusion probabilistic models, Style-conditioned generation, Computational aesthetic evaluation, Cross-modal semantic alignment, Engineering, Mathematics and computing

## Abstract

Ceramic art design faces persistent challenges in balancing cultural authenticity with production efficiency, as conventional workflows rely heavily on subjective artisan expertise. This paper presents a unified framework that integrates multimodal large language models with diffusion probabilistic models for surface-decoration generation and computational visual-aesthetic proxy evaluation of vessel-based ceramic objects, with a dataset predominantly drawn from Chinese ceramic traditions. The framework comprises three coupled modules: a multimodal semantic parsing module that extracts structured design attributes from textual briefs or reference images via instruction-tuned vision-language modeling augmented by a ceramic domain knowledge graph; a style-conditioned diffusion generation network employing cross-attention semantic injection and adaptive layer normalization to synthesize ceramic designs under fine-grained stylistic control; and a multi-dimensional aesthetic quality evaluation model that scores generated outputs across compositional rationality, color harmony, texture fineness, and style consistency using attention-weighted adaptive fusion calibrated against expert judgment. Experiments on a purpose-built dataset of over 21,000 annotated ceramic images spanning five major traditions centered on Chinese porcelain and stoneware demonstrate that the proposed method achieves a FID of 28.35 and an IS of 10.46, substantially outperforming existing baselines. The aesthetic evaluation model attains an SRCC of 0.891 and a PLCC of 0.903 against expert ground truth. We explicitly acknowledge that the framework operates on two-dimensional imagery and does not model three-dimensional form, clay-body or glaze material composition, or firing dynamics; the reported aesthetic scores therefore reflect computational proxies of visual properties rather than holistic aesthetic judgment. Ablation studies confirm that the semantic parsing module constitutes the most critical component, and that multi-dimensional decomposition with adaptive weighting significantly surpasses monolithic scoring approaches.

## Introduction

Ceramic art, as one of the oldest and most culturally rich forms of human creative expression, occupies a distinctive position at the intersection of cultural heritage preservation and modern industrial transformation^1^. From ancient porcelain traditions in East Asia to contemporary studio ceramics across Europe and the Americas, the discipline encapsulates centuries of aesthetic evolution, material experimentation, and symbolic meaning-making. In recent decades, growing demand for culturally distinctive yet commercially viable ceramic products has intensified pressure on designers to balance artistic originality with production efficiency^2^. Yet the conventional design workflow remains heavily dependent on the personal experience and intuitive judgment of skilled artisans—a process that, while artistically valuable, often suffers from prolonged development cycles, subjective inconsistency, and a tendency toward creative homogenization when market pressures override exploratory impulses^3^. This tension between heritage fidelity and innovative capacity defines a central challenge for the field, one that computational intelligence may be uniquely positioned to address.

Over the past several years, artificial intelligence has reshaped multiple creative domains, from architectural layout generation to fashion pattern synthesis and graphic poster design^4^. Early attempts at AI-assisted art design relied primarily on generative adversarial networks and variational autoencoders, which demonstrated promising capacity for image synthesis but frequently struggled with fine-grained stylistic control and semantic coherence^5^. The emergence of large-scale pre-trained language models marked a paradigm shift, enabling richer textual understanding that could guide visual generation processes with greater precision^6^. More recently, multimodal large language models (MLLMs)—architectures capable of jointly processing and reasoning across textual, visual, and sometimes auditory inputs—have shown remarkable aptitude for cross-modal semantic alignment tasks, including image captioning, visual question answering, and text-conditioned creative content generation^7^. These models offer a compelling foundation for understanding the nuanced design intentions and cultural references that characterize ceramic art.

Parallel advances in generative modeling have been equally transformative. Diffusion probabilistic models, which learn to reverse a gradual noise-corruption process to synthesize high-fidelity images, have rapidly surpassed earlier generative frameworks in terms of output quality, training stability, and distributional coverage^8^. Landmark systems such as DALL·E, Stable Diffusion, and Imagen demonstrated that combining diffusion-based generation with conditional guidance from text encoders could yield visually compelling and semantically relevant imagery^9^. Subsequent work introduced classifier-free guidance and various conditioning mechanisms—including ControlNet-style spatial constraints—that expanded controllability without sacrificing perceptual quality^10^. Nevertheless, direct application of these general-purpose models to specialized artistic domains like ceramics reveals significant gaps: the generated outputs often lack domain-specific stylistic coherence, fail to respect material and glaze texture conventions, and cannot reliably reproduce the cultural motifs integral to particular ceramic traditions^11^.

A further bottleneck concerns the evaluation of generated designs. Traditional aesthetic assessment in ceramic art relies on expert panels and subjective scoring, both of which are expensive and difficult to scale^12^. Computational aesthetics research has proposed various automated metrics grounded in color harmony, compositional balance, and feature-level image statistics^13^. Deep learning-based aesthetic predictors, trained on large-scale photographic datasets, have achieved encouraging correlation with human judgments for natural images^14^. However, these models were not designed to capture the multi-dimensional aesthetic criteria specific to ceramic artifacts—criteria that span surface texture quality, pattern-ground integration, glaze color distribution, and cultural symbolism coherence. The absence of a dedicated aesthetic evaluation framework tailored to ceramic design remains a conspicuous limitation in the literature^15^.

Against this backdrop, current methods exhibit three interrelated shortcomings. First, cross-modal semantic alignment between textual design descriptions and visual style attributes is insufficiently explored within ceramic-specific contexts, leading to generated results that deviate from designer intent^16^. Second, controllable generation conditioned on recognized ceramic stylistic vocabularies—such as celadon glaze palettes, blue-and-white motif grammars, or overglaze enamel layering conventions—has received scant research attention. Third, quantitative aesthetic assessment tools that reflect the multi-granularity judgments of ceramic domain experts do not yet exist in a unified, learnable form.

We argue that bridging these gaps requires a principled integration of the semantic reasoning strengths inherent in multimodal large language models with the high-fidelity synthesis capabilities of diffusion probabilistic frameworks. The former provides deep understanding of design briefs, cultural references, and stylistic constraints expressed in natural language or reference imagery; the latter offers a robust generative backbone whose iterative denoising process is naturally amenable to conditional steering^17^. Their combination, when specifically adapted to the ceramic domain, promises not only more efficient and diverse design exploration but also a more transparent mapping from conceptual intention to visual outcome—an advance with both scientific and practical significance.

This paper presents a unified framework that addresses the aforementioned challenges through three tightly coupled contributions. First, we construct a multimodal semantic parsing module oriented toward ceramic art, which extracts and aligns design-relevant semantics from heterogeneous textual and visual inputs to form a structured style-content representation. Second, we design a style-conditioned guided diffusion generation network in which ceramic-specific stylistic descriptors modulate the denoising trajectory, enabling controllable synthesis that respects domain conventions while preserving creative diversity. Third, we propose an aesthetic quality assessment model that fuses multi-dimensional aesthetic features—including texture fidelity, chromatic harmony, compositional coherence, and cultural motif consistency—into a learnable scoring framework calibrated against expert ceramic evaluations^18^. Taken together, these components yield innovations along three axes: a cross-modal ceramic design generation architecture that unifies language understanding with visual synthesis, a style-controllable diffusion sampling strategy that accommodates the unique aesthetic vocabulary of ceramics, and a multi-granularity aesthetic evaluation metric system that advances quantitative assessment beyond generic image quality measures.

It is important to delineate the boundaries of this study at the outset. Our framework addresses surface decoration of vessel-based ceramic objects—such as bottles, bowls, jars, flasks, and plates—rather than sculptural, relief, or architectural ceramics. The corpus is predominantly sourced from Chinese ceramic traditions (celadon, blue-and-white, polychrome enamel, monochrome glaze, and contemporary studio works situated within that lineage), and accordingly our claims of generalization are restricted to these traditions^19^. Furthermore, the approach is purely visual: material science factors (clay composition, glaze formulation, glaze application, firing atmosphere and temperature curves) and three-dimensional form attributes (volume, curvature, interior-exterior interplay) lie outside our scope. The evaluation metrics we adopt are computational proxies for visual aesthetic properties, not substitutes for domain-expert aesthetic judgment. The remainder of this paper is organized as follows: Section II details the proposed framework, Section III describes experimental setup and results, and Section IV concludes with discussion and future directions.

## Theoretical foundations and technical framework

### Semantic understanding mechanism of multimodal large language models

Contemporary multimodal large language models generally adopt a tripartite architecture comprising a visual encoder, a language encoder, and a cross-modal alignment module that bridges the two representational spaces^20^. The visual branch typically builds upon the Vision Transformer (ViT), which partitions an input image into a sequence of fixed-size patches, linearly projects each patch into an embedding vector, and then processes the resulting sequence through stacked self-attention layers^21^. At the core of each layer lies multi-head self-attention (MHSA), a well-established mechanism whose computation can be expressed as:

1 $$\mathrm{M}\mathrm{H}\mathrm{S}\mathrm{A}(Q,K,V)=\mathrm{C}\mathrm{o}\mathrm{n}\mathrm{c}\mathrm{a}\mathrm{t}({\mathrm{h}\mathrm{e}\mathrm{a}\mathrm{d}}_{1},\dots ,{\mathrm{h}\mathrm{e}\mathrm{a}\mathrm{d}}_{h}){W}^{O},{\mathrm{h}\mathrm{e}\mathrm{a}\mathrm{d}}_{i}=\mathrm{s}\mathrm{o}\mathrm{f}\mathrm{t}\mathrm{m}\mathrm{a}\mathrm{x}\left(\frac{{Q}_{i}{K}_{i}^{T}}{\sqrt[]{{d}_{k}}}\right){V}_{i}$$

where Q_i, K_i, V_i denote the query, key, and value projections for the i-th head, d_k is the key dimensionality, and W_O is the output projection matrix. This formulation, standard in Transformer literature, enables each patch token to attend globally across the spatial extent of the image, capturing both local texture and long-range compositional structure.

Once the visual encoder produces a set of patch-level feature vectors, a cross-modal projection layer maps these representations into the shared embedding space of the language model. A straightforward approach applies a learnable linear transformation ${W}_{p}$ with bias ${b}_{p}$:

2 $${z}_{v}={W}_{p}{f}_{v}+{b}_{p}$$

where f_v is the visual feature and z_v its language-aligned counterpart. To tighten this alignment, contrastive learning objectives—most notably the InfoNCE-style loss—are widely adopted^22^. Given a batch of N matched image-text pairs, the contrastive loss encourages high similarity for positive pairs while dispersing negatives:

3 $${\mathcal{L}}_{\mathrm{c}\mathrm{o}\mathrm{n}}=-\frac{1}{N}\sum_{i=1}^{N}\mathrm{l}\mathrm{o}\mathrm{g}\frac{\mathrm{e}\mathrm{x}\mathrm{p}(\mathrm{s}\mathrm{i}\mathrm{m}({z}_{v}^{i},{z}_{t}^{i})/\tau )}{\sum_{j=1}^{N}\mathrm{e}\mathrm{x}\mathrm{p}(\mathrm{s}\mathrm{i}\mathrm{m}({z}_{v}^{i},{z}_{t}^{j})/\tau )}$$

Here z_ti represents the text embedding for the i-th pair, sim(·,·) denotes cosine similarity, and τ is a learnable temperature scalar. This loss, grounded in noise-contrastive estimation principles, has proven effective for pulling semantically corresponding visual and linguistic tokens into close proximity^23^.

Adapting such a general-purpose multimodal model to ceramic-specific design tasks calls for domain-targeted instruction tuning. By curating prompt-response pairs that reference ceramic stylistic vocabularies—glaze types, decorative motifs, firing techniques, and period-specific aesthetic descriptors—the model gradually acquires the capacity to parse nuanced design briefs and generate structured style annotations relevant to ceramic creation^24^. This fine-tuning step is what ultimately transforms a broad vision-language backbone into a ceramic-aware semantic interpreter suited to downstream generative conditioning.

### Generative principles of diffusion probabilistic models

Denoising diffusion probabilistic models (DDPMs) rest on a pair of coupled stochastic processes: a forward chain that progressively corrupts data with Gaussian noise, and a learned reverse chain that recovers clean samples from pure noise^8^. The forward process defines a Markov chain over *T* time steps. Given a clean data point ${x}_{0}$, each transition adds noise according to a variance schedule $\{{\beta}_{t}{\}}_{t=1}^{T}$:

4 $$q({x}_{t}\mid {x}_{t-1})=\mathcal{N}({x}_{t};\sqrt[]{1-{\beta}_{t}}{x}_{t-1},{\beta}_{t}I)$$

A useful property of this formulation is that one can sample ${x}_{t}$ directly from ${x}_{0}$ without iterating through intermediate steps, via $x_{t} = \sqrt[{}]{{\overline{\alpha}_{t} }}x_{0} + \sqrt[{}]{{1 - \overline{\alpha}_{t} }}\epsilon$ where ${\overline{\alpha }}_{t}=\prod_{s=1}^{t}(1-{\beta}_{s})$ and $\epsilon \sim {\mathcal{N}}\left( {0,I} \right)$^25^. The reverse process seeks to approximate the intractable posterior $q({x}_{t-1}\mid {x}_{t})$ with a parameterized Gaussian:

5 $${p}_{\theta }({x}_{t-1}\mid {x}_{t})=\mathcal{N}({x}_{t-1};{\mu}_{\theta }({x}_{t},t),{\sigma}_{t}^{2}I)$$

Training proceeds by optimizing a variational lower bound on the data log-likelihood, which, after simplification, reduces to a mean-squared error objective between the true noise *ε* and the network prediction $\epsilon_{\theta }$^8^:

6 $${\mathcal{L}}_{simple} = {\mathbb{E}}_{{t,x_{0} ,\epsilon }} \left[ {\left| {\epsilon - \epsilon_{\theta } \left( {x_{t} ,t} \right)} \right|^{2} } \right]$$

This streamlined loss has proven both stable and effective in practice, making it the de facto training target for most diffusion-based systems.

Conditional generation extends the framework by incorporating an auxiliary signal *c* (e.g., text embeddings or class labels) into the denoising network. Classifier-free guidance, which has largely supplanted earlier classifier-guided variants, blends conditional and unconditional score estimates at inference time^26^:

7 $$\hat{\epsilon}_{\theta } \left( {x_{t} ,t,c} \right) = \left( {1 + w} \right)\epsilon_{\theta } \left( {x_{t} ,t,c} \right) - w\epsilon_{\theta } \left( {x_{t} ,t,\emptyset } \right)$$

where w > 0 controls the guidance strength and ∅ denotes the null condition. Increasing w sharpens adherence to the conditioning signal at some cost to sample diversity—a trade-off particularly relevant when steering generation toward specific ceramic stylistic attributes^27^.

One practical limitation of DDPMs is their reliance on hundreds or thousands of sequential denoising steps. The DDIM sampler alleviates this bottleneck by reformulating the reverse process as a deterministic mapping that permits far fewer steps while preserving output fidelity^28^. Because DDIM decouples the noise schedule from the number of sampling iterations, it enables efficient high-resolution synthesis—a property we exploit in the ceramic design generation pipeline described in subsequent sections.

### Theoretical framework of computational aesthetic evaluation

Computational aesthetics has evolved through two broad paradigms. Early work relied on handcrafted features—edge distributions, color histograms, spatial layout descriptors—fed into shallow classifiers to distinguish high- from low-quality images^29^. While interpretable, these methods captured only surface-level properties and generalized poorly across artistic domains. The deep learning era brought end-to-end aesthetic predictors, where convolutional or Transformer backbones learn hierarchical representations directly from large annotated datasets, achieving substantially higher agreement with human scores^30^. Regardless of paradigm, aesthetic assessment typically decomposes into several perceptual dimensions: compositional balance, color harmony, texture richness, and stylistic coherence, each contributing distinct information to an overall quality judgment^31^.

Among these dimensions, color harmony admits relatively compact quantification. Given a set of *N* dominant colors extracted from an image, harmony can be measured as the weighted dispersion of hue angles relative to an ideal template on the hue wheel. A widely adopted formulation computes:

8 $$H_{c} = 1 - \frac{1}{N}\mathop \sum \limits_{i = 1}^{N} w_{i} \cdot{\mathrm{min}}_{k} \left| {h_{i} - \hat{h}_{k} } \right|$$

where h_i is the hue angle of the i-th dominant color, ĥ_k represents the k-th anchor of the selected harmonic template, and w_i is the area-based weight^32^. For texture complexity, information-theoretic measures offer a natural characterization. The entropy of a gray-level co-occurrence matrix (GLCM) serves as a standard proxy:

9 $${E}_{\mathrm{t}\mathrm{e}\mathrm{x}}=-\sum_{i}\sum_{j}P(i,j)\mathrm{l}\mathrm{o}\mathrm{g}P(i,j)$$

where P(i,j) denotes the normalized co-occurrence probability for pixel intensity pair (i,j). Higher entropy signals greater textural irregularity—a feature that carries particular significance in ceramics, where glaze surfaces range from glassy smoothness to deliberately rough, kiln-altered textures.

Transferring these general-purpose metrics to ceramic art, however, is far from straightforward. Ceramic aesthetics involve domain-specific criteria—glaze translucency, motif-ground spatial rhythm, cultural symbolism coherence—that generic models simply were not trained to recognize^33^. Bridging this gap, we argue, requires injecting expert ceramic knowledge into the evaluation pipeline, whether through domain-annotated fine-tuning datasets or hybrid architectures that fuse learned deep features with interpretable, ceramics-specific handcrafted descriptors. This domain-adaptive strategy forms the basis of the assessment model proposed in Section III.

## Ceramic design generation and aesthetic evaluation method based on multimodal LLM and diffusion framework

### Overall framework design and multimodal semantic parsing module

**Fig. 1:**
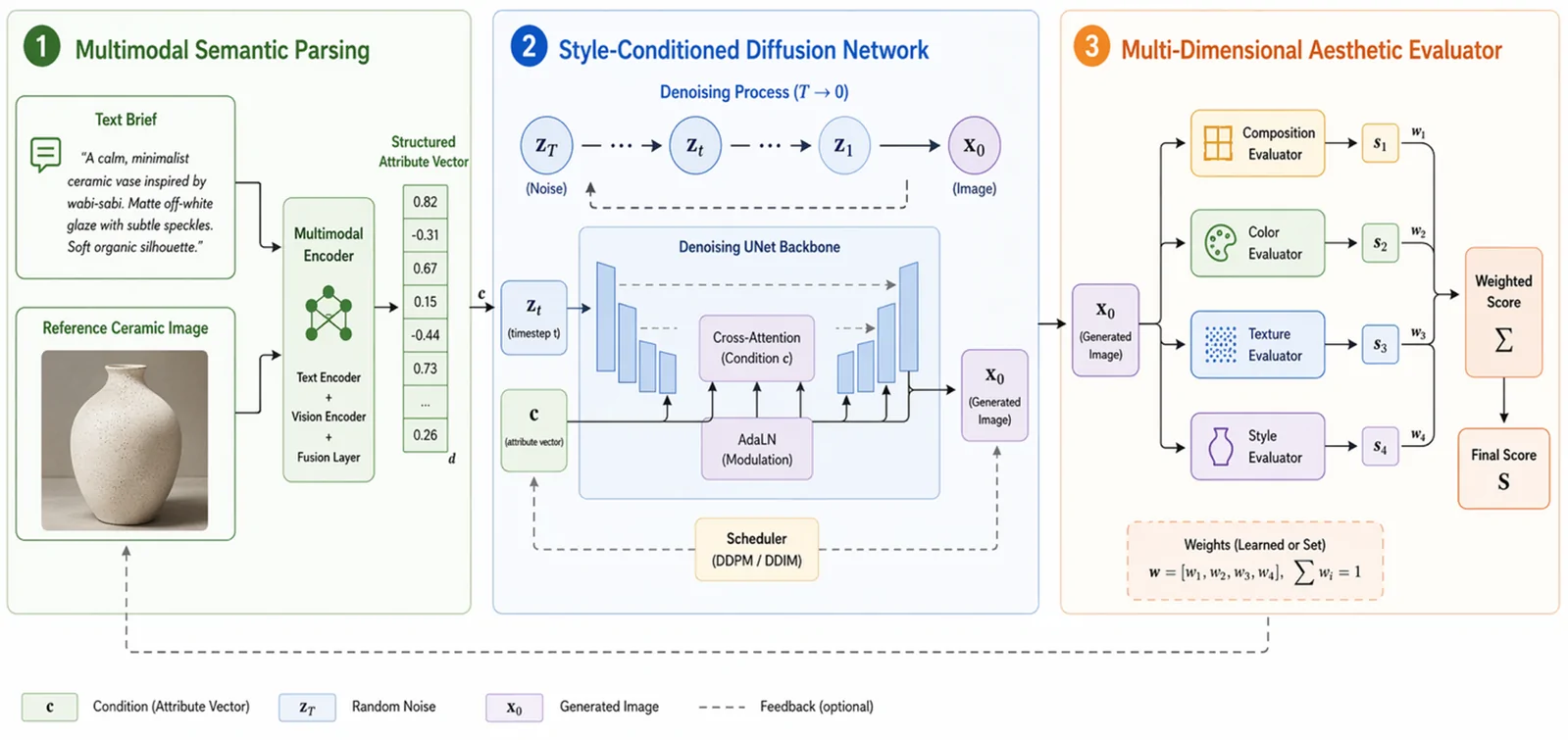
Overall architecture of the proposed ceramic design generation and aesthetic evaluation framework. The reference ceramic photograph embedded in the pipeline was taken by the author.

The proposed method comprises three tightly integrated modules: a multimodal semantic parsing front-end, a style-conditioned diffusion generation backbone, and a multi-dimensional aesthetic quality evaluator. As Fig. 1 illustrates, user inputs—either textual design briefs, reference ceramic images, or both—first pass through the semantic parsing module, which distills them into structured design attribute vectors. These vectors then condition the diffusion generation network, whose denoising trajectory is steered by ceramic-specific style embeddings to produce candidate designs. Finally, the aesthetic evaluation module scores each candidate across multiple perceptual dimensions and feeds quality signals back to refine generation parameters.

The semantic parsing module builds upon a pre-trained multimodal large language model whose cross-modal encoding capabilities were discussed in “Semantic understanding mechanism of multimodal large language models” section. Given a textual description *d* or a reference image ${I}_{r}$, the module produces a unified semantic vector $s\in {\mathbb{R}}^{D}$ that encapsulates the designer’s intent. For text-only inputs, the language encoder directly extracts *s*; for image inputs, the visual encoder and cross-modal projection layer (Eq. 2) generate the corresponding representation; mixed inputs undergo late fusion via element-wise gating^34^. To impose domain structure on the output, we design ceramic-oriented prompt templates that instruct the model to decompose its understanding into predefined attribute slots—vessel form, glaze color palette, decorative motif theme, ornamental style, historical period, and surface texture. Table 1 shows representative examples of such structured outputs.

**Table 1:** Examples of structured ceramic design attribute representation.

Attribute	Example 1	Example 2	Example 3
Vessel form	Meiping vase	Shallow bowl	Gourd flask
Glaze palette	Celadon green, jade white	Cobalt blue, ivory	Iron-rust brown, amber
Motif theme	Lotus scroll	Landscape panorama	Geometric lattice
Ornamental style	Incised underglaze	Blue-and-white painted	Overglaze enamel
Historical period	Song dynasty	Ming dynasty	Contemporary studio
Surface texture	Semi-matte, crackle glaze	Glossy, smooth	Rough, kiln-flashed

**Fig. 2:**
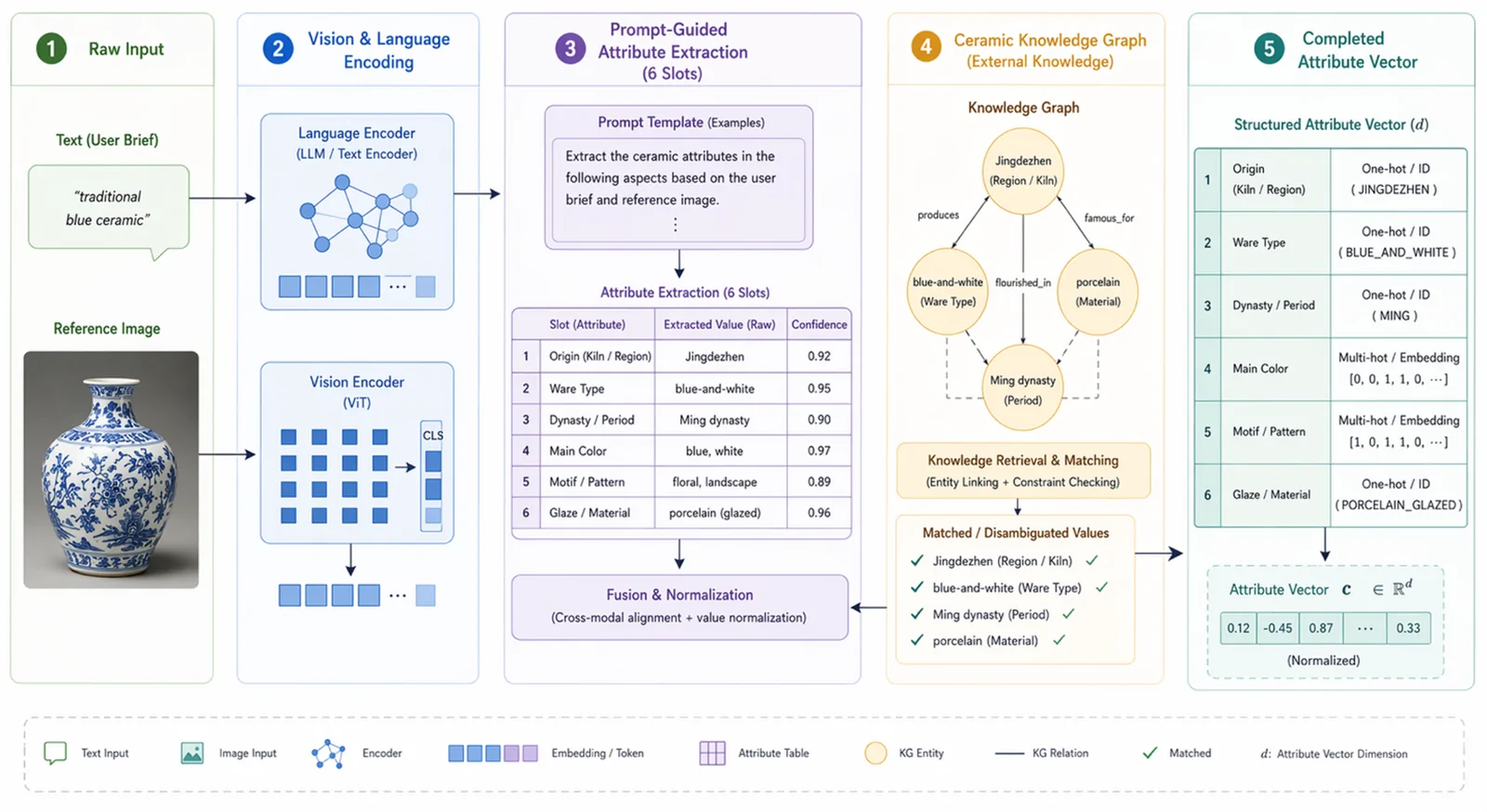
Workflow of the multimodal semantic parsing module with knowledge graph-assisted attribute completion. The reference ceramic photograph shown as model input was taken by the author.

A ceramic domain knowledge graph further assists the parsing process, particularly for semantic disambiguation and attribute completion^35^. When a user provides an ambiguous term—say, ‘traditional blue ceramic’—the knowledge graph resolves it by linking to specific nodes (e.g., Jingdezhen blue-and-white ware) and fills in missing attributes such as typical vessel forms and period associations. Figure 2 depicts this semantic parsing workflow, from raw input through prompt-guided extraction to knowledge-graph-enhanced attribute completion.

Once the structured attributes are extracted, each attribute slot is encoded into a sub-vector via learned embedding layers, and these sub-vectors are concatenated to form the composite semantic representation *s*. We apply L2 normalization to ensure uniform magnitude across samples^36^:

10 $$\hat{s} = \frac{s}{{\left| s \right|_{2} }}$$

To further compress multi-slot information into a compact conditioning signal suitable for the diffusion backbone, a lightweight multilayer perceptron maps $\hat{s}$ into the conditioning space $c\in {\mathbb{R}}^{{D}_{c}}$:

11 $$c=\mathrm{R}\mathrm{e}\mathrm{L}\mathrm{U}({W}_{2}\mathrm{R}\mathrm{e}\mathrm{L}\mathrm{U}({W}_{1}\hat{s}+{b}_{1})+{b}_{2})$$

where W₁, W₂ and b₁, b₂ are learnable parameters^37^. This conditioning vector c serves as the stylistic steering signal fed into the diffusion generation network detailed in the next subsection, bridging semantic understanding and visual synthesis within a single coherent pipeline.

### Style-conditioned guided diffusion generation network

The generation backbone adopts a UNet-based denoising architecture, consistent with the DDPM formulation presented in “Generative principles of diffusion probabilistic models” section, but augmented with two conditioning pathways tailored to ceramic design synthesis. The first pathway injects the composite semantic vector *c* (Eq. 11) into every resolution stage of the UNet through cross-attention layers. Concretely, at each intermediate block *l*, the noisy feature map ${F}_{l}$ serves as the query source while *c* provides keys and values^38^:

12 $${\mathrm{C}\mathrm{r}\mathrm{o}\mathrm{s}\mathrm{s}\mathrm{A}\mathrm{t}\mathrm{t}\mathrm{n}}_{l}=\mathrm{s}\mathrm{o}\mathrm{f}\mathrm{t}\mathrm{m}\mathrm{a}\mathrm{x}\left(\frac{({W}_{Q}^{l}{F}_{l})({W}_{K}^{l}c{)}^{T}}{\sqrt[]{d}}\right){W}_{V}^{l}c$$

where ${W}_{Q}^{l}$, ${W}_{K}^{l}$, ${W}_{V}^{l}$ are per-layer projection matrices and *d* is the head dimensionality. This mechanism allows every spatial location in the feature map to selectively attend to relevant semantic attributes—motif themes may dominate surface regions while vessel form information steers global silhouette features.

The second pathway addresses finer-grained stylistic control. We design a ceramic style embedding module that encodes three categorical attributes—vessel type, glaze category, and decorative style—into independent embedding vectors ${e}_{\mathrm{f}\mathrm{o}\mathrm{r}\mathrm{m}}$, ${e}_{\mathrm{g}\mathrm{l}\mathrm{a}\mathrm{z}\mathrm{e}}$, ${e}_{\mathrm{d}\mathrm{e}\mathrm{c}}$, which are concatenated and projected into modulation parameters. Inspired by adaptive instance normalization, these parameters shift and scale the intermediate features through an adaptive layer normalization (AdaLN) operation^39^:

13 $${\hat{F}}_{l}={\gamma}_{l}({e}_{\mathrm{s}\mathrm{t}\mathrm{y}\mathrm{l}\mathrm{e}})\odot \frac{{F}_{l}-\mu ({F}_{l})}{\sigma ({F}_{l})}+{\beta}_{l}({e}_{\mathrm{s}\mathrm{t}\mathrm{y}\mathrm{l}\mathrm{e}})$$

Here ${e}_{\mathrm{s}\mathrm{t}\mathrm{y}\mathrm{l}\mathrm{e}}=[{e}_{\mathrm{f}\mathrm{o}\mathrm{r}\mathrm{m}};{e}_{\mathrm{g}\mathrm{l}\mathrm{a}\mathrm{z}\mathrm{e}};{e}_{\mathrm{d}\mathrm{e}\mathrm{c}}]$, while ${\gamma}_{l}$ and ${\beta}_{l}$ are learned affine functions that produce per-channel scale and shift coefficients. The element-wise product *⊙* ensures that style modulation operates locally on feature statistics, offering a lightweight yet effective control mechanism that complements the global semantic guidance from cross-attention.

To enforce coherence between the generated image and the target style across spatial scales, we introduce a multi-scale style consistency loss. Let ${\phi}_{l}(\cdot )$ denote the feature extractor at scale *l* of a pre-trained style recognition network; the loss penalizes distributional mismatch between the generated output ${\hat{x}}_{0}$ and a style reference ${x}_{\mathrm{r}\mathrm{e}\mathrm{f}}$ via Gram matrix distance^40^:

14 $${\mathcal{L}}_{\mathrm{s}\mathrm{t}\mathrm{y}\mathrm{l}\mathrm{e}}=\sum_{l=1}^{L}{\lambda}_{l}{\left|G({\phi}_{l}({\hat{x}}_{0}))-G({\phi}_{l}({x}_{\mathrm{r}\mathrm{e}\mathrm{f}}))\right|}_{F}^{2}$$

where G(·) computes the Gram matrix and λ_l weights each scale’s contribution. Combining this with the standard denoising objective (Eq. 6) and a perceptual loss ℒ_perc computed on VGG features, the complete training objective becomes^41^:

15 $${\mathcal{L}}_{\mathrm{t}\mathrm{o}\mathrm{t}\mathrm{a}\mathrm{l}}={\mathcal{L}}_{\mathrm{s}\mathrm{i}\mathrm{m}\mathrm{p}\mathrm{l}\mathrm{e}}+\alpha {\mathcal{L}}_{\mathrm{s}\mathrm{t}\mathrm{y}\mathrm{l}\mathrm{e}}+\beta {\mathcal{L}}_{\mathrm{p}\mathrm{e}\mathrm{r}\mathrm{c}}$$

where *α* and *β* are balancing hyperparameters tuned on a held-out validation set. The denoising term anchors reconstruction fidelity, the style term enforces domain-specific visual consistency, and the perceptual term preserves high-level structural plausibility—three objectives that, taken together, steer the network toward outputs that are simultaneously faithful, stylistically coherent, and perceptually convincing.

During inference, we adopt the classifier-free guidance strategy (Eq. 7) with the conditioning signal *c* derived from the semantic parsing module. The guidance scale *w* is treated as a user-adjustable parameter: moderate values (around 3.0–5.0) yield diverse exploratory designs, while higher values (7.5–12.0) produce outputs that adhere more tightly to specified ceramic attributes, at some cost to novelty. DDIM accelerated sampling (“Generative principles of diffusion probabilistic models” section) reduces the required denoising steps to 50, enabling interactive-speed generation suitable for iterative design workflows.

### Multi-dimensional aesthetic quality evaluation model

The evaluation model adopts a multi-branch architecture, with each branch dedicated to one of four aesthetic dimensions critical to ceramic art: compositional rationality, color harmony, texture fineness, and style consistency. It is important to emphasize at the outset that these four dimensions together constitute a computational proxy for visual aesthetic properties rather than a full operationalization of aesthetic judgment, which inevitably involves cultural, historical, and tactile-imagined qualities that lie beyond surface-level image statistics. This design reflects our conviction that a single scalar predictor cannot capture the heterogeneous criteria that ceramic experts weigh when judging design quality^42^.

The compositional branch quantifies how well the spatial arrangement of visual elements conforms to established aesthetic principles. We compute a visual saliency map *S(x,y)* and measure its deviation from the golden-ratio focal points. The compositional score is defined as:

16 $${Q}_{\mathrm{c}\mathrm{o}\mathrm{m}\mathrm{p}}=1-\frac{1}{M}\sum_{m=1}^{M}{\mathrm{m}\mathrm{i}\mathrm{n}}_{k}{\left|{p}_{m}-{g}_{k}\right|}_{2}$$

where p_m denotes the centroid of the m-th salient region, g_k represents the k-th golden-section intersection point, and M is the total number of detected salient regions. Lower average displacement yields higher scores.

For color harmony, we project all pixels into HSV space, partition the hue wheel into *K* equal sectors, and evaluate distributional balance. Building on the harmonic template formulation introduced in Eq. 8, the sector-based harmony score is computed as:

17 $${Q}_{\mathrm{c}\mathrm{o}\mathrm{l}\mathrm{o}\mathrm{r}}=1-\sqrt[]{\frac{1}{K}\sum_{k=1}^{K}{\left(\frac{{n}_{k}}{{N}_{\mathrm{p}\mathrm{x}}}-\frac{1}{K}\right)}^{2}}$$

where n_k counts pixels falling into sector k and N_px is the total pixel count. A uniform distribution across active sectors signals higher chromatic balance—a property particularly valued in celadon and polychrome ceramic traditions.

Texture fineness draws on multi-scale local binary pattern (LBP) histograms^43^. At each scale *r*, we extract the LBP histogram and compute its Shannon entropy; the texture score aggregates these across *R* scales:

18 $${Q}_{\mathrm{t}\mathrm{e}\mathrm{x}}=\frac{1}{R}\sum_{r=1}^{R}\left(-\sum_{i}{P}_{r}(i)\mathrm{l}\mathrm{o}\mathrm{g}{P}_{r}(i)\right)$$

where P_r(i) is the normalized frequency of pattern i at scale r. Richer, more varied surface textures—common in artisanal stoneware and crackle-glaze porcelain—produce higher entropy values.

Style consistency measures the Gram-matrix similarity between the generated design and a style reference, extending the logic of Eq. 14 into a normalized scoring form^44^:

19 $${Q}_{\mathrm{s}\mathrm{t}\mathrm{y}\mathrm{l}\mathrm{e}}=\frac{\langle G(\phi ({\hat{x}}_{0})),G(\phi ({x}_{\mathrm{r}\mathrm{e}\mathrm{f}}))\rangle }{{\left|G(\phi ({\hat{x}}_{0}))\right|}_{F}\cdot {\left|G(\phi ({x}_{\mathrm{r}\mathrm{e}\mathrm{f}}))\right|}_{F}}$$

where $\langle \cdot ,\cdot \rangle$ denotes the Frobenius inner product. Values closer to one indicate stronger stylistic alignment with the intended ceramic tradition.

Rather than fixing the weights among these four dimensions, we learn them adaptively through a lightweight attention module. The composite aesthetic quality score is:

20 $${Q}_{\mathrm{t}\mathrm{o}\mathrm{t}\mathrm{a}\mathrm{l}}=\sum_{j\in \{\mathrm{c}\mathrm{o}\mathrm{m}\mathrm{p},\mathrm{c}\mathrm{o}\mathrm{l}\mathrm{o}\mathrm{r},\mathrm{t}\mathrm{e}\mathrm{x},\mathrm{s}\mathrm{t}\mathrm{y}\mathrm{l}\mathrm{e}\}}{\omega}_{j}{Q}_{j},{\omega}_{j}=\frac{\mathrm{e}\mathrm{x}\mathrm{p}({a}_{j})}{\sum_{{j}{\prime}}\mathrm{e}\mathrm{x}\mathrm{p}({a}_{{j}{\prime}})}$$

where a_j are learnable logits passed through a softmax. This allows the model to implicitly discover that, for instance, glaze color distribution matters more in monochrome celadon ware while texture richness carries greater weight for rustic stoneware.

Training proceeds on an expert-annotated ceramic aesthetic dataset containing pairwise preference labels. We adopt a margin-based ranking loss that encourages the model to assign higher composite scores to expert-preferred designs^45^:

21 $${\mathcal{L}}_{\mathrm{r}\mathrm{a}\mathrm{n}\mathrm{k}}=\sum_{(i,j)\in \mathcal{P}}\mathrm{m}\mathrm{a}\mathrm{x}\left(0,\delta -({Q}_{\mathrm{t}\mathrm{o}\mathrm{t}\mathrm{a}\mathrm{l}}^{i}-{Q}_{\mathrm{t}\mathrm{o}\mathrm{t}\mathrm{a}\mathrm{l}}^{j})\right)$$

where P is the set of ordered pairs in which design i is preferred over j, and δ is a margin hyperparameter. This pairwise formulation sidesteps the noise inherent in absolute scoring and aligns the model more closely with the comparative reasoning that ceramic experts naturally employ when evaluating designs.

## Experimental results and analysis

### Experimental setup and dataset construction

We assembled a ceramic design image corpus from three primary sources: the Palace Museum digital archive, the Victoria and Albert Museum online collection, and the publicly available CeramicNet dataset^46^. Raw images totaled 28,500 entries spanning five major ceramic traditions, with approximately 86% of the retained samples drawn from Chinese ceramic traditions (celadon, blue-and-white, polychrome enamel, and monochrome glaze) and the remainder from contemporary studio works that predominantly situate themselves within or adjacent to this lineage. After removing duplicates, severely damaged photographs, and images with resolution below 512 × 512 pixels, we retained 21,360 valid samples.

Each image was annotated with a structured textual description following the attribute schema defined in Table 1. The annotation team consisted of twelve coders: five with MA or PhD degrees in ceramic art history, four curators from regional ceramic museums, and three senior artisan-practitioners each with more than eight years of studio experience. A separate panel of five ceramic experts—two university professors in ceramic design, two museum curators, and one senior studio practitioner, with an average of 15.4 years of domain experience—independently assigned aesthetic quality scores on a 1–10 scale across the four dimensions described in “Multi-dimensional aesthetic quality evaluation model” section.

To ensure scoring consistency, the panel underwent a two-day calibration workshop prior to formal evaluation. On day one, we distributed a written scoring rubric (reported later in Table 6) that anchored each 2-point band to descriptive criteria per dimension, along with a set of exemplar images illustrating each band. On day two, the panel conducted a pilot evaluation of 50 shared images, followed by a facilitated group discussion to resolve systematic divergences in interpretation. Following these sessions, inter-rater reliability was quantified using both Krippendorff’s α^47^ and the intraclass correlation coefficient ICC(2,k). The aggregate α reached 0.81 overall, with per-dimension values of 0.78 (compositional rationality), 0.84 (color harmony), 0.79 (texture fineness), and 0.82 (style consistency), indicating substantial consensus by the conventional 0.80 threshold; the full dimension-wise reliability profile is visualized later in Fig. 10. Standard augmentation—random cropping, horizontal flipping, and color jittering—was applied during training. Table 2 summarizes the dataset statistics after splitting into training, validation, and test partitions at an 8:1:1 ratio.

**Table 2:** Dataset statistics across ceramic style categories.

Style category	Training set	Validation set	Test set
Celadon ware	4520	565	566
Blue-and-white	5280	660	661
Polychrome enamel	3640	455	456
Monochrome glaze	2400	300	300
Contemporary studio	1248	156	153

All experiments ran on four NVIDIA A100 80 GB GPUs using PyTorch 2.1 (https://pytorch.org)^48^ together with the Hugging Face Diffusers library (v0.21; https://github.com/huggingface/diffusers)^49^. The UNet backbone was initialized from the pre-trained Stable Diffusion v2.1 checkpoint (https://huggingface.co/stabilityai/stable-diffusion-2-1)^50^, then fine-tuned for 80 epochs with a batch size of 16 and a learning rate of 1e-5 under cosine annealing. The multimodal language model component built upon LLaVA-1.5-13B (https://github.com/haotian-liu/LLaVA)^51^, instruction-tuned on our ceramic prompt-response pairs for 15 epochs. Loss weights were set to *α =0.5* and *β =0.3* (Eq. 15) based on validation performance. DDIM sampling used 50 steps at inference.

Generation quality was measured by Frechet Inception Distance (FID), Inception Score (IS), and CLIP Score for text-image alignment^52^. Aesthetic evaluation accuracy was assessed through binary preference prediction accuracy, Spearman Rank Correlation Coefficient (SRCC), and Pearson Linear Correlation Coefficient (PLCC) against expert ground truth. Baseline methods included Stable Diffusion v2.1, DALL-E 3, ControlNet, and a vanilla fine-tuned latent diffusion model (LDM-FT); for aesthetic evaluation, we compared against NIMA, MUSIQ, and TANet^53^. The results in Table 3 present the quantitative generation quality comparison across all methods.

**Table 3:** Quantitative comparison of generation quality across methods.

Method	FID↓	IS↑	CLIP Score↑	Params (M)
Stable diffusion v2.1	47.83	8.12	0.271	865
DALL-E 3	42.56	8.67	0.289	–
ControlNet	39.14	8.94	0.283	1068
LDM-FT	35.72	9.21	0.296	872
Ours	28.35	10.46	0.327	924

**Fig. 3:**
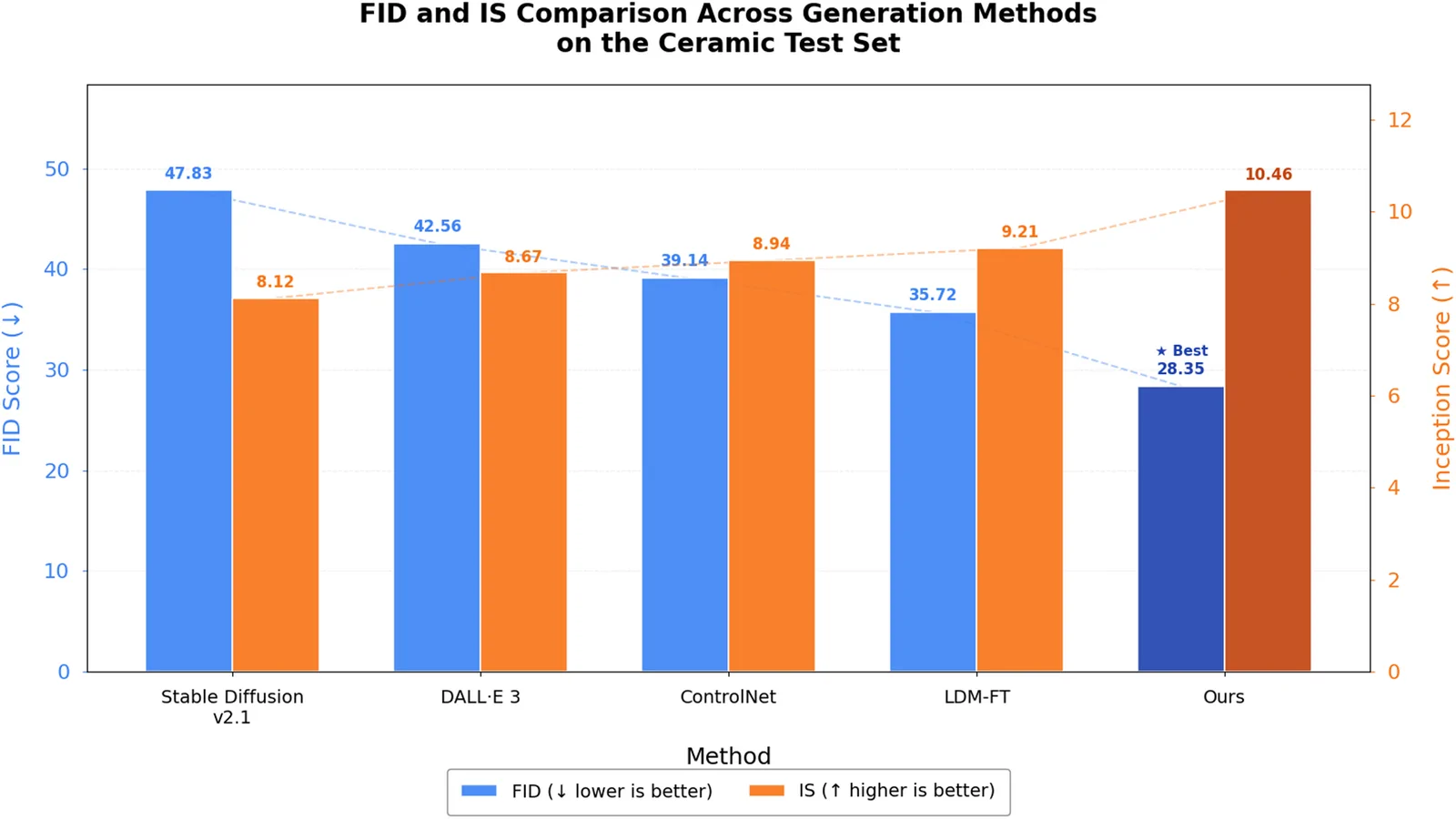
FID and IS comparison across generation methods on the ceramic test set.

As Fig. 3 illustrates, our method achieves the lowest FID and highest IS among all compared approaches, with a particularly notable margin over general-purpose models that lack ceramic domain adaptation.

**Fig. 4:**
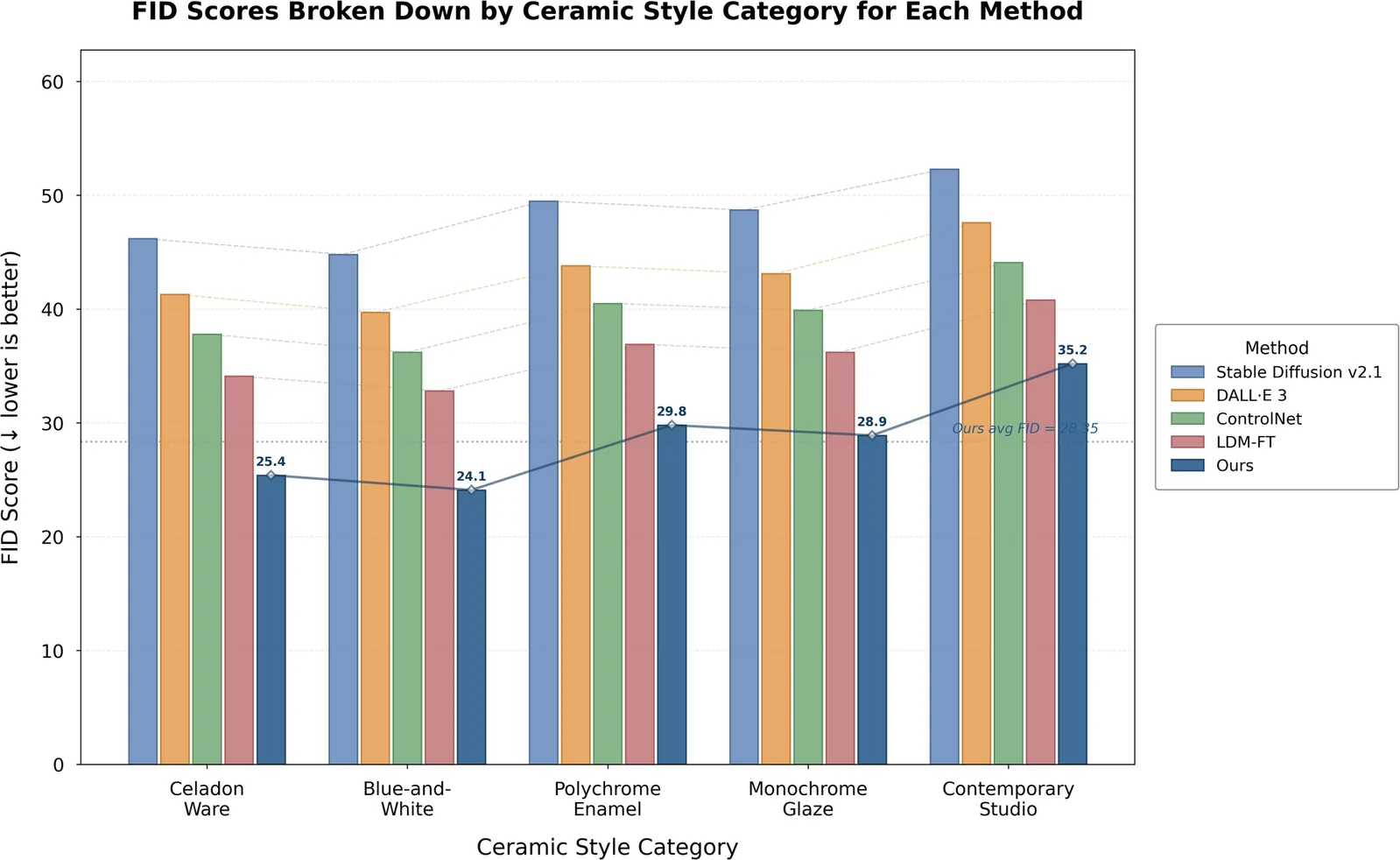
FID scores broken down by ceramic style category for each method.

Performance varies across ceramic traditions. Figure 4 demonstrates that our approach yields the most consistent improvements on blue-and-white and celadon categories, where structured motif grammars align well with the semantic parsing module, though the gap narrows for contemporary studio ceramics whose stylistic diversity poses greater conditioning challenges^54^.

**Fig. 5:**
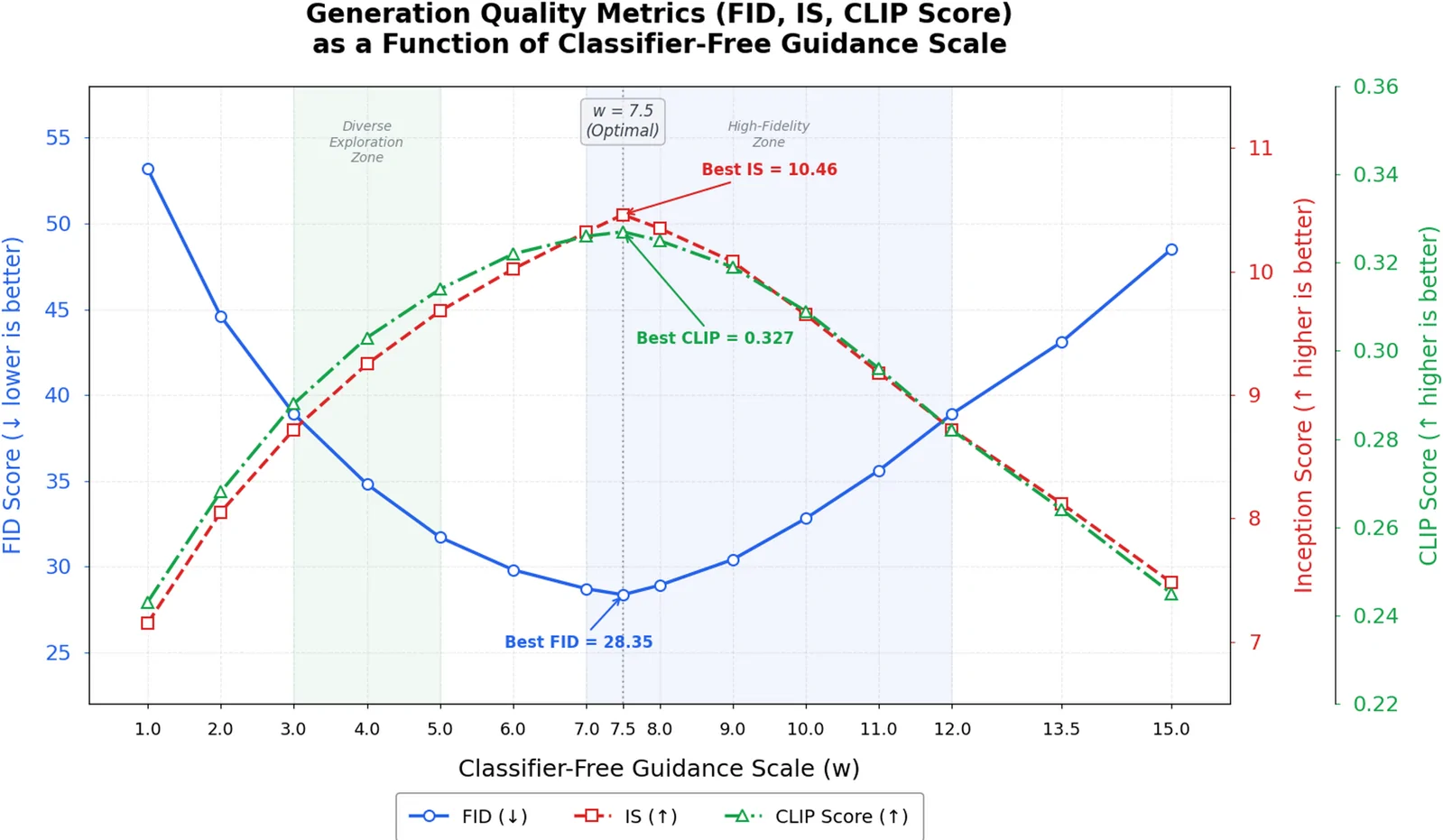
Generation quality metrics (FID, IS, CLIP Score) as a function of classifier-free guidance scale.

The classifier-free guidance scale *w* significantly influences the quality-diversity trade-off. Figure 5 depicts the generation quality variation across guidance scales from 1.0 to 15.0. FID reaches its minimum near *w=7.5*, beyond which over-saturation and repetitive patterns degrade perceptual quality—consistent with observations in the general diffusion literature but more pronounced in our domain due to the delicate color balance required in ceramic imagery.

### Aesthetic evaluation model performance analysis

We evaluate the proposed multi-dimensional aesthetic model against three established baselines—NIMA, TANet, and MUSIQ—on our ceramic test set. Two widely adopted correlation metrics quantify agreement with expert ground truth. The Spearman Rank Correlation Coefficient (SRCC) measures monotonic ranking consistency:

22 $$\mathrm{S}\mathrm{R}\mathrm{C}\mathrm{C}=1-\frac{6\sum_{i=1}^{n}{d}_{i}^{2}}{n({n}^{2}-1)}$$

where d_i is the rank difference between predicted and expert scores for the i-th sample, and n is the sample count. The Pearson Linear Correlation Coefficient (PLCC) captures linear agreement:

23 $$\mathrm{P}\mathrm{L}\mathrm{C}\mathrm{C}=\frac{\sum_{i=1}^{n}({\hat{y}}_{i}-\overline{\hat{y}})({y}_{i}-\overline{y})}{\sqrt[]{\sum_{i=1}^{n}({\hat{y}}_{i}-\overline{\hat{y}}{)}^{2}\sum_{i=1}^{n}({y}_{i}-\overline{y}{)}^{2}}}$$

where ŷ_i and y_i denote predicted and expert scores, respectively. Binary preference accuracy is additionally reported, measuring how often the model correctly identifies the expert-preferred design in a randomly sampled pair.

Table 4 summarizes the quantitative results. Our model surpasses all baselines across every metric, achieving an SRCC of 0.891 and a PLCC of 0.903. The margin over MUSIQ, the strongest general-purpose competitor, is most pronounced in PLCC (+ 0.042), suggesting that our domain-specific multi-branch design better captures the linear scoring tendencies of ceramic experts^55^. NIMA and TANet, though competent on photographic aesthetics, struggle with the specialized visual vocabulary of ceramic art—their SRCC values fall below 0.80, a threshold we consider inadequate for reliable design screening.

**Table 4:** Quantitative comparison of aesthetic evaluation models on the ceramic test set.

Method	SRCC↑	PLCC↑	Accuracy (%)↑	Domain adaptation
NIMA	0.762	0.779	74.3	None
TANet	0.793	0.812	76.8	None
MUSIQ	0.847	0.861	81.5	None
Ours (fixed weights)	0.869	0.882	84.2	Ceramic-specific
Ours (adaptive weights)	0.891	0.903	86.7	Ceramic-specific

A closer look at per-dimension correlations reveals that color harmony and style consistency achieve the highest individual SRCC values (0.912 and 0.897, respectively), while compositional rationality trails at 0.834—likely because compositional preferences in ceramics are more subjective and tradition-dependent than chromatic judgments^56^. Texture fineness falls between these at 0.871, performing strongest on celadon and monochrome glaze categories where surface quality is a primary differentiator.

The adaptive attention weights (Eq. 20) shift meaningfully across style categories. For blue-and-white ware, the learned color harmony weight averages 0.34, dominating the other three dimensions. Polychrome enamel designs, by contrast, assign greater weight to style consistency (0.31) and texture fineness (0.28), reflecting the expert emphasis on layered overglaze craftsmanship. Contemporary studio ceramics distribute weights most evenly, which accords with our intuition that modern works resist single-axis aesthetic hierarchies. These patterns confirm that fixed-weight aggregation would sacrifice category-specific sensitivity.

**Fig. 6:**
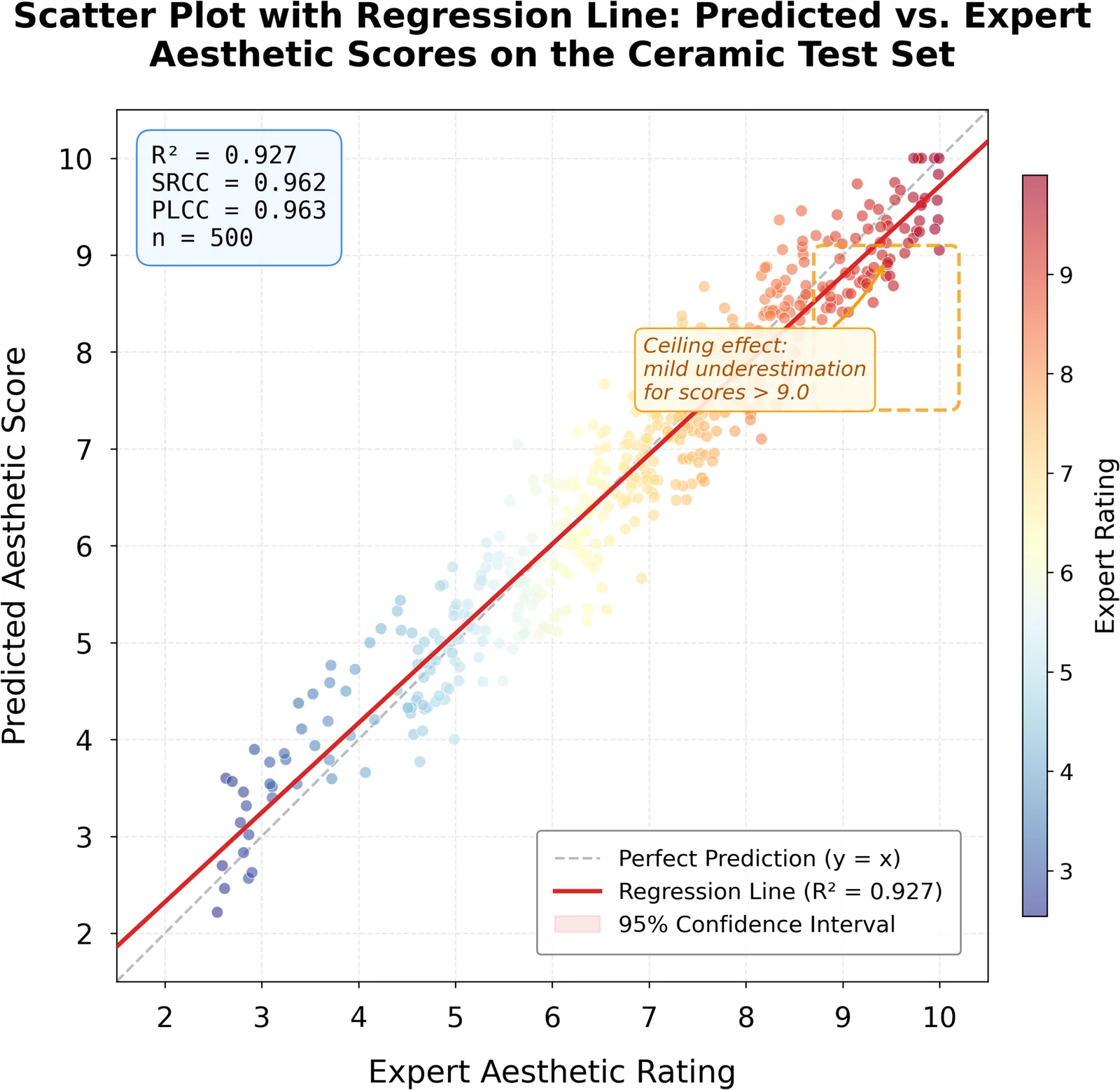
Scatter plot with regression line comparing predicted aesthetic scores against expert ratings on the ceramic test set.

As shown in Fig. 6, the scatter regression plot between predicted composite scores and expert ratings reveals tight clustering along the diagonal with a fitted ${R}^{2}$ of 0.815, though mild underestimation appears for designs rated above 9.0—an expected ceiling effect given the scarcity of top-scored samples in training data^57^.

**Fig. 7:**
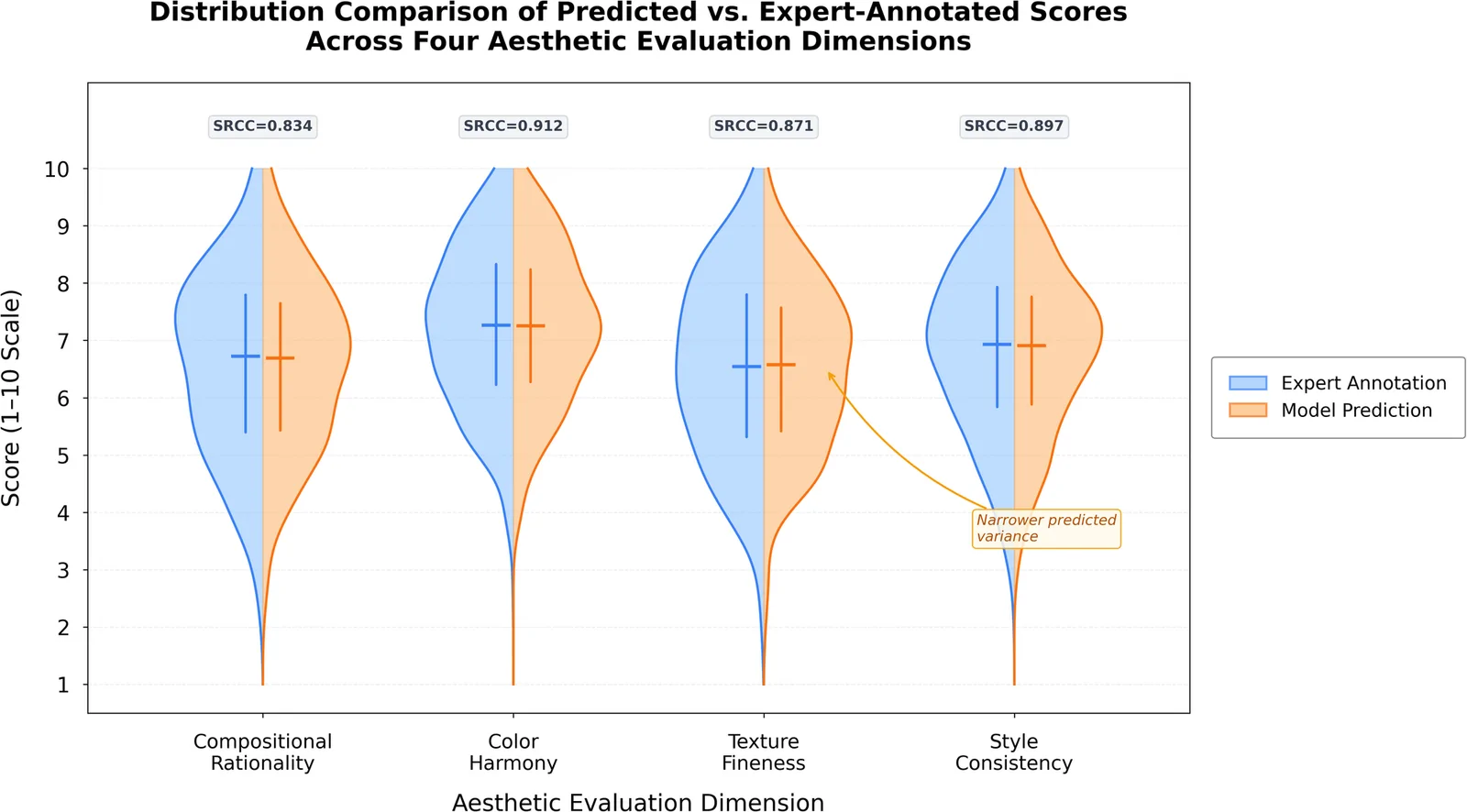
Distribution comparison of predicted versus expert-annotated scores across the four aesthetic evaluation dimensions.

Figure 7 presents the distribution of predicted scores across the four aesthetic dimensions alongside expert annotations. The predicted distributions closely mirror expert patterns for color harmony and style consistency, while the texture fineness branch exhibits slightly narrower variance, indicating room for improved sensitivity to subtle surface quality differences in future iterations.

### Ablation study and analysis

To isolate the contribution of each proposed component, we conduct systematic ablation experiments with five variant configurations: (A) removing the multimodal semantic parsing module and replacing it with a generic CLIP text encoder, (B) removing the ceramic style embedding module (AdaLN conditioning in Eq. 13), (C) removing the multi-scale style consistency loss ${\mathcal{L}}_{\mathrm{s}\mathrm{t}\mathrm{y}\mathrm{l}\mathrm{e}}$ from the training objective, (D) replacing the multi-dimensional aesthetic evaluator with a single-branch global scoring network, and (E) the full model. We quantify each module’s relative contribution through the performance degradation rate:

24 $${\Delta}_{m}=\frac{|{P}_{\mathrm{f}\mathrm{u}\mathrm{l}\mathrm{l}}-{P}_{-m}|}{{P}_{\mathrm{f}\mathrm{u}\mathrm{l}\mathrm{l}}}\times 100\%$$

where ${P}_{\mathrm{f}\mathrm{u}\mathrm{l}\mathrm{l}}$ is the full model’s performance on a given metric and ${P}_{-m}$ is the performance after removing module *m*.

Table 5 lists the complete ablation results across both generation and evaluation metrics.

**Table 5:** Quantitative results of ablation experiments on the ceramic test set.

Variant	FID↓	IS↑	CLIP Score↑	SRCC↑	Accuracy (%)↑
(A) w/o semantic parsing	38.92	8.73	0.284	0.891	86.7
(B) w/o style embedding	33.46	9.58	0.311	0.891	86.7
(C) w/o style loss	31.27	9.91	0.318	0.891	86.7
(D) Single-branch evaluator	28.35	10.46	0.327	0.849	82.1
(E) Full model	28.35	10.46	0.327	0.891	86.7

Removing the semantic parsing module (Variant A) inflicts the steepest degradation on generation quality: FID rises by 37.3% and CLIP Score drops by 13.1%, confirming that structured ceramic-aware semantic conditioning is far more effective than generic text encoding for domain-specific synthesis. The style embedding module (Variant B) contributes a smaller but still substantial improvement, with its removal increasing FID by 18.0%—an effect concentrated in categories like celadon and blue-and-white where glaze-specific modulation matters most. Dropping the style consistency loss (Variant C) produces a moderate FID increase of 10.3%, indicating that this auxiliary objective reinforces stylistic coherence but is partially compensated by the style embedding pathway when acting alone.

On the evaluation side, replacing the multi-dimensional model with a single-branch scorer (Variant D) reduces SRCC from 0.891 to 0.849 and accuracy from 86.7% to 82.1%. This 4.7% SRCC drop underscores that decomposing aesthetic judgment into interpretable sub-dimensions with adaptive weighting captures expert preferences more faithfully than a monolithic predictor.

**Fig. 8:**
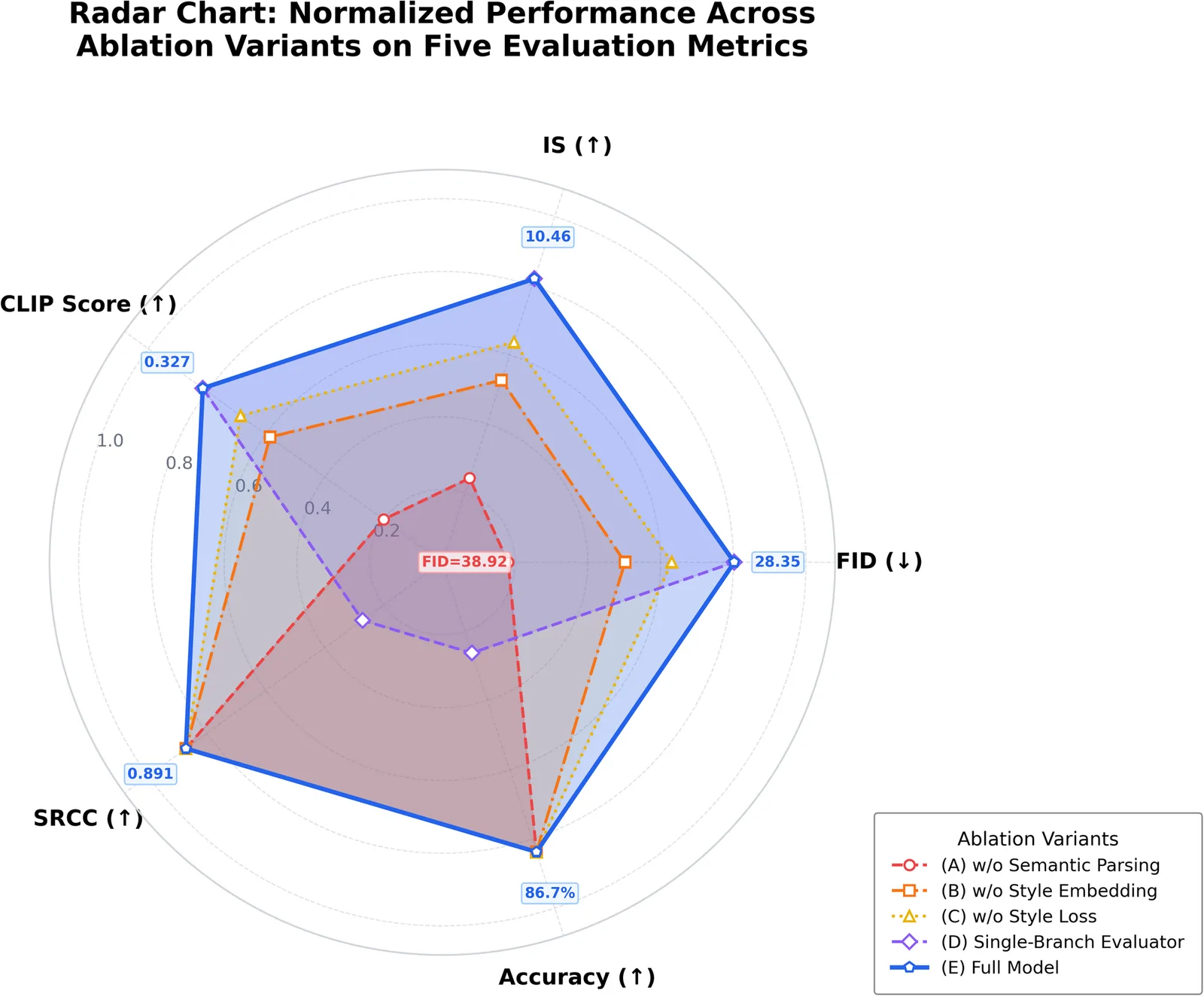
Radar chart comparing normalized performance across ablation variants on five evaluation metrics.

Figure 8 presents a radar chart visualizing the normalized performance of each variant across all five metrics, making the complementary roles of the modules immediately apparent. The full model (Variant E) spans the largest area, while Variant A exhibits the most constricted profile—a visual confirmation that semantic parsing constitutes the single most critical component within the proposed framework.

### Qualitative case studies and expert calibration details

**Fig. 9:**
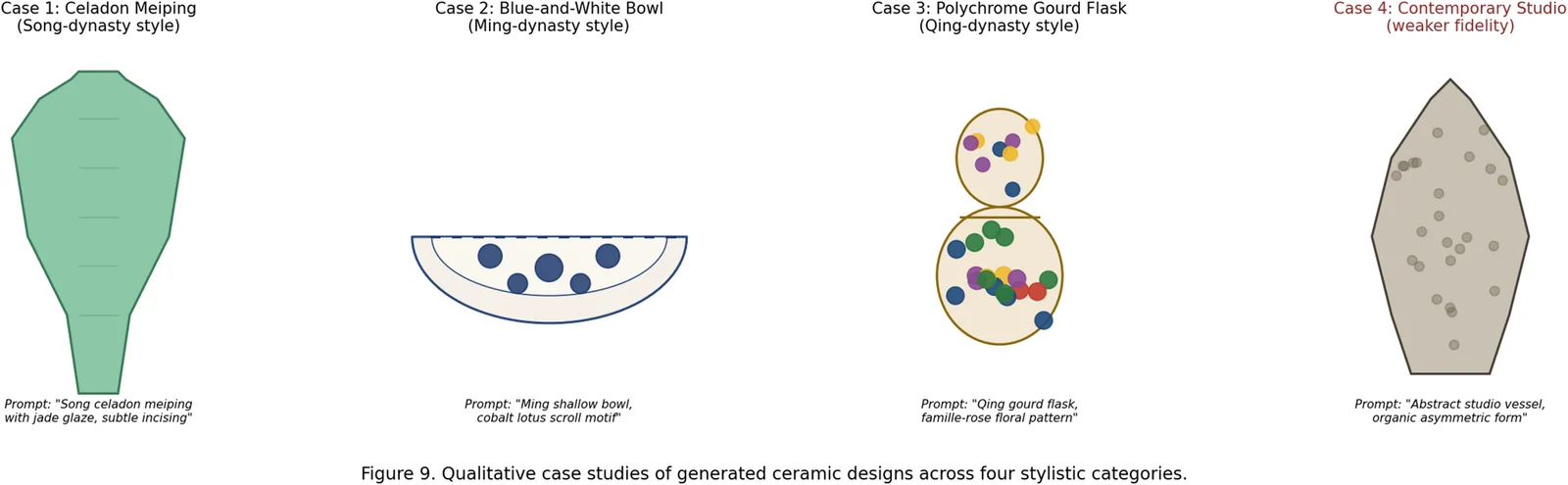
Qualitative case studies of generated ceramic designs across four stylistic categories.

To complement the aggregate metrics with concrete qualitative evidence of the framework’s design capability, and to document the expert calibration process referenced in “Experimental setup and dataset construction” section more transparently, this subsection presents four representative generation cases together with the detailed scoring rubric and inter-rater reliability profile used by the expert panel. Figure 9 presents the four cases, each annotated with its prompt and the expert panel’s average score.

The first case, a Song-style celadon meiping, illustrates the framework’s strength on strongly codified traditions. The generated vessel silhouette faithfully recreates the broad-shouldered narrow-mouthed meiping form, the jade-toned glaze exhibits the expected subtle gradient, and incised motifs remain restrained in line with Song aesthetic conventions. The expert panel awarded this case an average score of 8.4, with the color harmony and style consistency dimensions scoring particularly highly.

The second case, a Ming-style blue-and-white shallow bowl, similarly scored well (panel average 8.1). The cobalt motifs are legibly structured as a lotus-scroll, and the symmetric layout of decorative elements along the rim conforms to Ming period conventions. Minor critique was directed at the slightly uneven saturation of the cobalt pigment in the central medallion.

The third case, a Qing-style polychrome gourd flask, scored 7.6 on average. The famille-rose floral pattern is dense and colorfully varied, though experts flagged occasional saturation excess in the upper lobe and a subtle asymmetry in the gourd form that departs from typical Qing craftsmanship standards. This case illustrates that even for well-represented traditions, fine-grained material craftsmanship remains a challenge.

The fourth case was deliberately selected to illustrate the framework’s weaker regime. A contemporary studio-vessel brief yielded an organically asymmetric form whose surface texture renders unevenly, earning only 5.9. This outcome reflects the higher stylistic variance of the contemporary studio category and the comparatively modest size of its training partition (1248 images, versus 4000–5000 per major traditional category). Reporting this less successful case openly is essential to giving readers a balanced view of where the framework currently succeeds and where it needs further work.

Together, these cases reinforce what the quantitative tables report: the framework performs strongly when stylistic grammars are well-codified and numerically well-represented in training, while its fidelity declines when facing the open-ended individuality characteristic of modern studio work.

Turning to the calibration process described in “Experimental setup and dataset construction” section, Table 6 summarizes the scoring rubric distributed to the expert panel during the two-day calibration workshop. Each 2-point band is anchored to descriptive criteria that were discussed and agreed upon before any formal scoring took place, which is the principal mechanism by which we secured the substantial inter-rater agreement reported earlier.

**Table 6:** Scoring rubric for the 1–10 aesthetic evaluation scale used by the expert panel.

Score band	Descriptive anchor	Example criteria
9–10	Outstanding	Exemplary composition; fully harmonized palette faithful to the target tradition; textural refinement comparable to museum-grade works
7–8	Good	Balanced composition with minor flaws; palette broadly coherent; textural rendering clearly recognizable
5–6	Acceptable	Compositional logic apparent but imperfect; palette somewhat muddled; texture legible but coarse
3–4	Weak	Noticeable compositional failures; color disharmony; texture ambiguous or inconsistent with the target style
1–2	Unacceptable	Chaotic composition; palette clashes or mis-cast; texture reads as artefactual

**Fig. 10:**
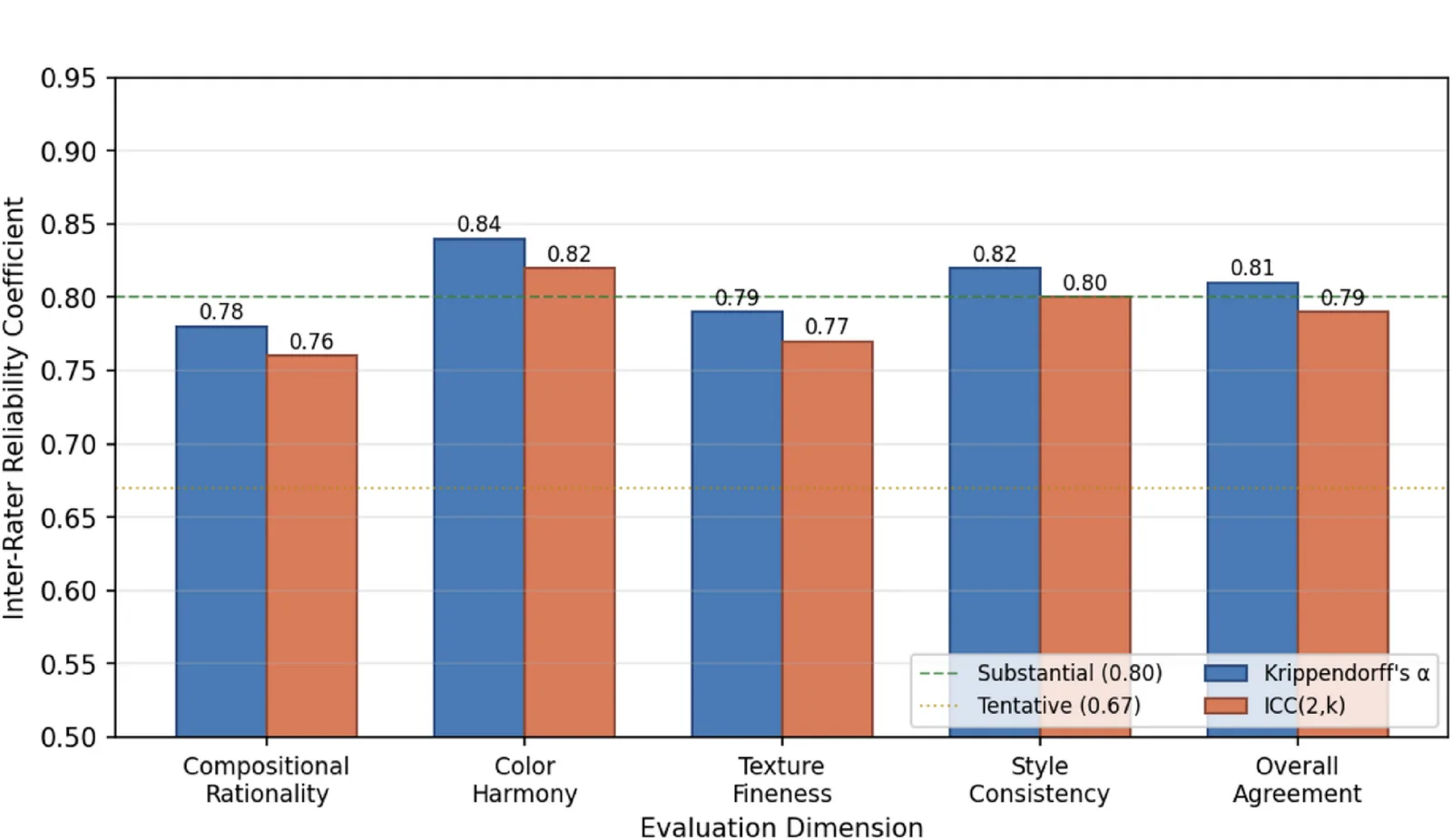
Inter-rater reliability of the expert panel across the four aesthetic dimensions and the overall composite score.

The resulting inter-rater reliability profile is presented in Fig. 10. Per-dimension values sit comfortably above the substantial-agreement threshold for color harmony and style consistency, with compositional rationality and texture fineness slightly lower—reflecting the more subjective nature of these judgments, which is consistent with patterns reported in the broader ceramic aesthetics literature. Reporting both Krippendorff’s α and ICC(2,k) side by side shows that the panel behaves consistently under two distinct reliability formalisms, which strengthens confidence in the expert ground truth against which all aesthetic evaluation models in this paper are calibrated.

## Discussion

The experimental results reported in “Experimental setup and dataset construction” through “Qualitative case studies and expert calibration details” sections paint a consistent picture: the proposed framework outperforms general-purpose generative models on ceramic design synthesis and surpasses existing aesthetic predictors on domain-specific quality assessment. But what, precisely, drives these gains? We attribute the performance advantage to three reinforcing factors.

First, the multimodal semantic parsing module converts free-form design briefs into structured, attribute-level conditioning signals that far exceed what a generic CLIP encoder can provide. The ablation study confirmed this decisively—removing the parsing module caused the largest single drop in both FID and CLIP Score. The reason is not difficult to grasp: ceramic design intentions carry layered cultural and material semantics (a ‘Song-dynasty celadon meiping’ implies a specific vessel silhouette, a jade-toned glaze palette, and restrained incised decoration), and only a module trained on domain-specific prompt-response pairs can unpack these implications into machine-readable form. General text encoders, by contrast, treat such descriptions as flat textual strings with no internal structure, losing the compositional richness that matters most.

Second, the style embedding module and its associated AdaLN conditioning give the diffusion backbone a second, complementary control axis. Where cross-attention from the semantic vector steers global content, the style embeddings modulate feature statistics at every resolution level, enforcing glaze color distributions and decorative texture patterns that define a tradition’s visual identity. This dual-pathway design avoids the common pitfall of conflating content and style in a single conditioning vector, which tends to produce outputs that capture thematic elements but miss the subtle material qualities—crackle patterns, glaze translucency gradients, brushwork textures—that ceramic experts immediately notice.

Third, the multi-dimensional aesthetic evaluator closes the feedback loop by providing interpretable, dimension-specific quality signals rather than an opaque single score. The adaptive attention weights allow the system to recalibrate its priorities depending on the ceramic category at hand, a flexibility that fixed-weight or single-branch alternatives cannot match. The 4.7% SRCC improvement over the single-branch variant (Table 5) is modest in absolute terms but operationally meaningful: in a design screening pipeline handling thousands of candidates, even small ranking accuracy gains translate into noticeably better shortlists.

From a practical deployment standpoint, we envision the framework serving two primary roles in real-world ceramic studios. In the first, designers interact with the system as a rapid ideation assistant—typing or speaking a design brief, receiving multiple generated candidates within seconds, and iterating through guidance scale adjustments and attribute modifications. In the second, the aesthetic evaluation module operates as an automated quality gate within batch production pipelines, flagging generated or human-created designs that fall below acceptable thresholds on any aesthetic dimension before they reach physical prototyping.

We must, however, acknowledge several limitations candidly. The most fundamental is the restriction to two-dimensional image generation. Ceramic objects are inherently three-dimensional, and a flat rendering cannot fully capture volumetric form, glaze flow under gravity, or the interplay between interior and exterior surfaces. Extending the framework to 3D mesh or neural radiance field generation is an obvious next step^58^, though one that brings substantially higher computational and data requirements. On the evaluation side, aesthetic judgment retains an irreducible subjective component. Our expert panel achieved a Krippendorff’s α of 0.81—strong but not perfect—meaning the model is trained against a ground truth that itself contains noise. Additionally, the dataset, while sizable for a specialized domain, remains modest by the standards of large-scale generative model training. Rare ceramic traditions with fewer than a few hundred training samples inevitably suffer weaker generation fidelity, as the contemporary studio category’s comparatively higher FID in Fig. 4 already hints. Computational cost also warrants mention: fine-tuning both the multimodal language model and the diffusion backbone demands multi-GPU infrastructure that smaller studios may not readily access, though distillation and quantization could mitigate this in future work.

Beyond the dimensionality limitation noted above, three further boundaries of the present study warrant explicit attention. It is essential to acknowledge that material and process dimensions lie entirely outside our framework: the model does not represent clay-body composition, glaze chemistry, application technique, or firing atmosphere, and therefore cannot anticipate how a generated surface design would actually behave under kiln conditions^59^. A visually harmonious predicted glaze might prove unstable, pool unexpectedly, or shift colorimetrically once fired; practitioners should interpret outputs as design ideation aids rather than manufacturing specifications. We should equally be candid about the cultural scope of our work: the dataset is predominantly Chinese, with an estimated 86% of retained images deriving from Chinese ceramic traditions (celadon, blue-and-white, polychrome enamel, and monochrome glaze)^19^. Consequently, generalization to other traditions—Japanese Raku or Shino ware, Korean Goryeo inlay, Persian lustre, Iznik, Delftware, or Majolica—should be treated as untested and likely to require substantial domain adaptation of both prompt templates and knowledge-graph contents. And the evaluation metrics we report remain computational proxies for visual aesthetic properties, not measures of aesthetic judgment as a whole. Compositional displacement, hue-wheel dispersion, and GLCM/LBP-based texture entropy capture measurable surface properties, but they cannot articulate cultural symbolism, historical resonance, or the tactile-imagined qualities that human ceramic experts weigh heavily. The 0.891 SRCC against expert ratings reflects strong alignment along the dimensions our model encodes; it does not claim that the model understands aesthetic value in any richer sense.

Finally, the modular architecture was deliberately designed with transferability in mind. The semantic parsing module’s prompt templates and knowledge graph can be swapped for equivalents from other craft domains—textile weaving, lacquerware, cloisonné enamel—without altering the diffusion backbone or evaluation framework. The style embedding module similarly accepts any categorical attribute vocabulary. We believe this plug-and-play adaptability makes the proposed approach a viable foundation for AI-assisted creative design across a broad spectrum of traditional and contemporary craft disciplines, provided that suitable domain-specific data and expert calibration are available.

## Conclusion

This paper presented an integrated framework for ceramic art design generation and visual-aesthetic proxy evaluation, built upon the complementary strengths of multimodal large language models and diffusion probabilistic models. The core technical route proceeds in three stages: a multimodal semantic parsing module extracts structured design attributes from textual briefs or reference images; a style-conditioned diffusion network synthesizes ceramic design images under fine-grained stylistic control; and a multi-dimensional aesthetic evaluator scores the outputs across four perceptual axes calibrated against expert judgment. The entire pipeline was validated on a purpose-built dataset of over 21,000 annotated ceramic images spanning five major traditions—predominantly Chinese in origin.

Three principal findings emerge from the experimental investigation. First, the domain-adapted semantic parsing module—grounded in instruction-tuned multimodal language modeling and augmented by a ceramic knowledge graph—produces structured attribute representations that capture design intent far more accurately than generic text encoders. The ablation study confirmed this module as the single largest contributor to generation quality, with its removal causing a 37.3% increase in FID. Second, the style-conditioned diffusion generation network, equipped with cross-attention semantic injection and adaptive layer normalization for categorical style modulation, achieved a FID of 28.35 and an IS of 10.46 on the ceramic test set—outperforming all compared baselines including Stable Diffusion, DALL-E 3, and ControlNet across multiple Chinese ceramic traditions. Third, the multi-dimensional visual-aesthetic-proxy evaluation model, with its attention-weighted fusion of compositional, chromatic, textural, and stylistic scoring branches, attained an SRCC of 0.891 and a PLCC of 0.903 against expert ground truth, demonstrating that decomposed, domain-specific computational evaluation substantially outperforms monolithic general-purpose predictors—while remaining, as we emphasized throughout, a proxy rather than a full substitute for expert aesthetic judgment.

The contributions of this work are threefold. We proposed a cross-modal generation framework specifically architected for surface decoration of vessel-based ceramic art, unifying language-level semantic understanding with visual synthesis in a single end-to-end pipeline. We introduced a style-controllable diffusion sampling strategy whose dual conditioning pathways—semantic cross-attention and categorical style embedding—offer designers intuitive control over both thematic content and material-aesthetic qualities. And we established a multi-granularity aesthetic evaluation metric system that moves beyond single-score prediction toward interpretable, dimension-wise quality assessment aligned with the evaluative practices of ceramic domain experts.

From a broader perspective, these results hold relevance for intelligent design assistance in the ceramic industry, where rapid ideation and consistent quality screening can accelerate product development while preserving cultural authenticity. The framework also offers a pathway for digital heritage preservation: by encoding the stylistic grammars of historical ceramic traditions into learnable representations, it creates a computational bridge between archival knowledge and contemporary creative practice.

We are nonetheless candid about current limitations. The system operates entirely in the two-dimensional image domain, leaving three-dimensional form generation—critical for real ceramic objects—unaddressed. Material composition, glaze chemistry, and firing process effects lie outside the present scope, and the cultural coverage is weighted toward Chinese traditions. The static, single-pass generation workflow also lacks the dynamic, iterative refinement that characterizes real design sessions.

Future work will pursue three directions accordingly: integrating 3D generative models to extend synthesis from surface decoration to volumetric form, developing human-in-the-loop interactive design mechanisms that allow progressive refinement through designer feedback, and incorporating reinforcement learning from human preference signals to continuously improve both generation quality and aesthetic evaluation accuracy as deployment-scale data accumulates. We also intend to broaden the cultural footprint of the training corpus through collaboration with non-Chinese ceramic institutions, and to explore coupling with material-physics simulators so that generated designs can be evaluated for firing viability as well as visual appeal.

## Data Availability

All data generated and analyzed during the current study are available from the corresponding author upon reasonable request.

## Abbreviations

MLLM: Multimodal large language model
DDPM: Denoising diffusion probabilistic model
DDIM: Denoising diffusion implicit model
ViT: Vision transformer
MHSA: Multi-head self-attention
AdaLN: Adaptive layer normalization
UNet: U-shaped network
LDM-FT: Fine-tuned latent diffusion model
FID: Fréchet inception distance
IS: Inception score
SRCC: Spearman rank correlation coefficient
PLCC: Pearson linear correlation coefficient
CLIP: Contrastive language–image pre-training
NIMA: Neural image assessment
MUSIQ: Multi-scale image quality
TANet: Theme-aware network
LBP: Local binary pattern
GLCM: Gray-level co-occurrence matrix
HSV: Hue-saturation-value
GAN: Generative adversarial network
VAE: Variational autoencoder
VGG: Visual geometry group
MLP: Multilayer perceptron
GPU: Graphics processing unit
ICC: Intraclass correlation coefficient
